# Density-Dependent Effects of Simultaneous Root and Floral Herbivory on Plant Fitness and Defense

**DOI:** 10.3390/plants12020283

**Published:** 2023-01-07

**Authors:** Martin Aguirrebengoa, Caroline Müller, Peter A. Hambäck, Adela González-Megías

**Affiliations:** 1 Departamento de Zoología, Universidad de Granada, 18071 Granada, Spain; 2 Department of Chemical Ecology, Bielefeld University, 33615 Bielefeld, Germany; 3 Department of Ecology, Environment and Plant Sciences, Stockholm University, 106 91 Stockholm, Sweden

**Keywords:** Brassicaceae, resistance, tolerance, compensation, density-dependence, plant defense, belowground–aboveground, insect herbivory, semiarid, transgenerational effects

## Abstract

Plants are attacked by multiple herbivores, and depend on a precise regulation of responses to cope with a wide range of antagonists. Simultaneous herbivory can occur in different plant compartments, which may pose a serious threat to plant growth and reproduction. In particular, plants often face co-occurring root and floral herbivory, but few studies have focused on such interactions. Here, we investigated in the field the combined density-dependent effects of root-chewing cebrionid beetle larvae and flower-chewing pierid caterpillars on the fitness and defense of a semiarid Brassicaceae herb. We found that the fitness impact of both herbivore groups was independent and density-dependent. Increasing root herbivore density non-significantly reduced plant fitness, while the relationship between increasing floral herbivore density and the reduction they caused in both seed number and seedling emergence was non-linear. The plant defensive response was non-additive with regard to the different densities of root and floral herbivores; high floral herbivore density provoked compensatory investment in reproduction, and this tolerance response was combined with aboveground chemical defense induction when also root herbivore density was high. Plants may thus prioritize specific trait combinations in response to varying combined below- and aboveground herbivore densities to minimize negative impacts on fitness.

## 1. Introduction

Herbivory imposes strong selection pressure on plants to replace or minimize tissue loss and to prevent subsequent reductions in fitness [1,2]. In response to herbivory, plants have developed diverse resistance and tolerance mechanisms, involving reconfigurations of the primary and secondary metabolism [3,4,5]. Often, resistance and tolerance are partially expressed together because concurrently allocating resources to both defense strategies can be more than additive [6,7,8,9]. Under natural conditions, plants are attacked simultaneously by multiple herbivores, and the cost of defense and the damage caused by a given herbivore may constrain a plant’s ability to resist or tolerate damage from other herbivores [10,11,12,13,14]. Nonetheless, extensive reviews and meta-analyses have shown that the combined effects of different herbivore groups on plant reproduction are prevalently independent [11,15]. When simultaneous herbivory occurs on different parts of the plant, the impact on reproduction is likely to be additive [15], although this assumption is mostly based on folivores and suckers as aboveground herbivores [15,16]. From the plant perspective, whether multiple herbivore effects are independent or not mostly depends on the capacity to compensate damage, the plant parts consumed, and the temporal concurrence of herbivory [15]. From the herbivore perspective, it mostly depends on the herbivore density (or intensity of herbivory), and the strength of the direct or indirect interactions between herbivores [15].

Herbivore density and the interactions among herbivore groups play a determinant role in plant defense evolution [17]. Because tolerance, resistance, and attack intensity are linked, it is necessary to simultaneously quantify these three parameters to decipher the relationships among them. Herbivore effects can be density-dependent, that is, the effects vary with their density. Examples abound in the case of large herbivorous mammals, in which the non-linear relationship between herbivore density/intensity and plant damage is well established [18,19,20,21,22]. Density-dependent effects may also occur in response to insect herbivory, although in this case the existing studies are scarcer. Some studies have demonstrated the occurrence of density-dependent effects of insect herbivory on plant tolerance or resistance not only in single but also in dual herbivore species scenarios [23,24,25,26].

Root and floral herbivory are among the most damaging types of herbivory for plants [16,27,28]. Root herbivory can decrease water and nutrient uptake and thus reduce the rates of photosynthesis. Removal of belowground biomass usually has larger effects on plant fitness than removal of an equivalent proportion of aboveground biomass [16]. To deal with root herbivores, plants display diverse resistance and tolerance responses, such as increased chemical defense, regrowth of lost roots, and nutrient allocation from roots to aboveground tissues [29,30,31,32]. Florivory, namely the consumption of ephemeral and/or immature plant reproductive structures, is receiving increasing attention due to the generally negative fitness impact of the consumption of these costly tissues [27,33,34,35]. Plants may be under selection to decrease feeding by floral herbivores and/or mitigate the fitness costs associated with floral damage [27,36,37,38,39,40,41]. Plants can opt for increased chemical defenses against floral herbivores, although the success of this strategy against specialized herbivores may depend on their counteradaptations [7,42,43] and on the eventual effects on the pollinator visitation rates [35]. Compensatory responses to florivory, by producing more flowers or by shunting resources to future flowers after damage, can also occur [27,44,45]. Tolerance to florivory is even more likely than for folivory because resource sinks (reproductive tissues) instead of resource sources are consumed [27,33]. 

Through systemic induction of chemical defenses aboveground [29,46], and nutrient allocation to reproductive tissues [30], root herbivores can affect aboveground herbivore performance and damage on plants [47,48,49,50]. Less known are the effects of floral herbivores on herbivores belowground and their joint effects on plants. Although probably very common in nature, few studies have investigated the effects of simultaneous root and floral herbivory [49,51], and even fewer have quantified their transgenerational effects in plants [52]. Herbivore-induced transgenerational effects such as changes in seedling emergence on offspring occur through alterations in maternal seed provisioning and/or modifications in inherited gene expression regulation between other mechanisms [53,54,55,56,57,58]. Because it exerts a strong selective pressure, accounting for potential transgenerational effects in short-lived plants’ emergence is crucial for an optimal fitness quantification [53,59,60,61,62]. None that we know of has studied the within-generational and transgenerational effects of simultaneous root and floral herbivory in a density-dependence framework.

In the wild Brassicaceae species *Moricandia moricandioides*, root-chewing by a single beetle larva and naturally occurring flower-chewing by pierid caterpillars was found to independently affect plant defense and reproduction, but only florivory was detrimental to plant fitness in terms of seed yield and seedling emergence on offspring [51,52]. However, the effect of these two herbivore groups can be density-dependent [16,27,33]. Thus, it remains to be unraveled whether herbivore damage, plant tolerance and resistance, and ultimately plant fitness, additively or non-additively vary according to the different density combinations of the two herbivore groups. With this aim, we carried out an experiment on the *M. moricandioides* system, in which the densities of both root herbivores and floral herbivores were manipulated in the field in a density gradient. Several plant traits related to growth, defense, and reproduction were measured, as well as the seedling emergence on offspring. Given that the caterpillar performance and growth rate determine the damage they cause on plants [63,64,65,66,67,68], we also evaluated the floral herbivore caterpillar development time in the different treatments. We tested whether (i) the plant defense responses to both herbivore group densities would be independent, (ii) the impact on plant fitness of both herbivore group densities would be additive, and (iii) the impact on plant fitness of both herbivore groups would linearly rather than non-linearly increase with their density.

## 2. Results

### 2.1. Development of Plant Reproduction over Time (Generalized Estimating Equations—GEEs)

**Figure 1:**
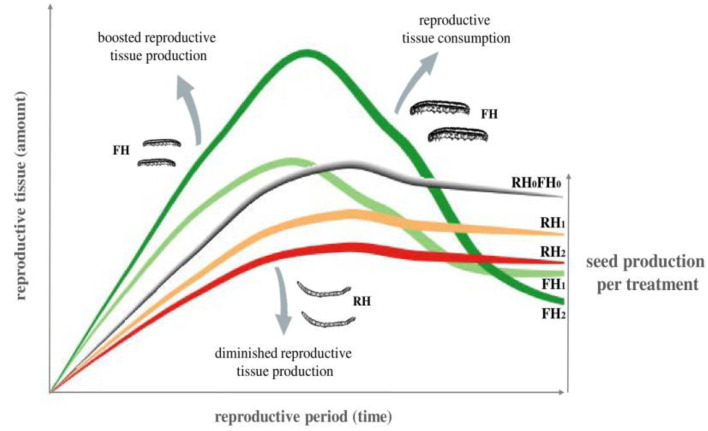
Illustrative representation of the temporal dynamics of plant reproductive tissue production and reproductive tissue loss throughout the reproductive period up to the number of seeds produced when interacting with different densities (0—absence, 1—low, 2—high) of root herbivores (RH) and floral herbivores (FH). Left side y axis encompasses the general pattern observed for the reproductive tissues: floral bud groups, flowers, and fruits.

A higher density of the root herbivores (RH) decreased floral bud group and fruit production on *M. moricandioides* but had no effect on flower production over time (Table 1; see Figure 1 for an illustrative approximation). A higher density of floral herbivores (FH) instead enhanced flower and fruit production but had no effect on floral bud production over time (Table 1; see Figure 1). No interactive effects of RH and FH densities on reproductive tissue production were observed over time (Table 1).

### 2.2. Linear and Non-Linear Effects of Herbviores on Plant Performance and Reproduction

GAMMs (generalized additive mixed models) performed better than GLMMs (general or generalized linear mixed models) for tests involving the number of flowers, fruits, and seeds (Supplementary Material 1, Table S1); in all the cases, the non-linear effect occurred because low and high densities of RH, FH, or both had a similar effect. GLMMs performed better in the rest of the cases: when effects were linear, when there was a sign change in the effect according to the density of RH or FH, and when interactive effects of RH and FH density were observed (Supplementary Material 1, Table S1).

**Figure 2:**
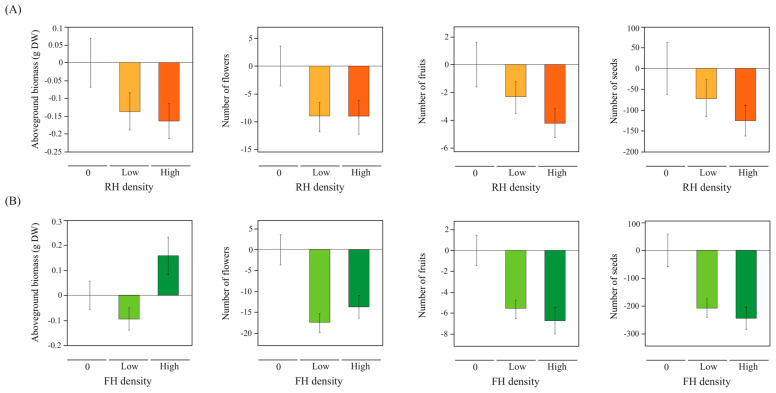
(**A**) Root herbivore (RH) density effects on plant aboveground biomass and reproductive traits. Shown values (mean ± SE) are relative to mean RH_0_ = 0, plants with no RH (see absolute values in Supplementary Material 2, Figure S1). White bars are shown for 0 RH density (RH_0_ plants, absence of RH), orange bars are shown for low RH density (RH_1_ plants, one RH individual), red bars are shown for high RH density (RH_2_ plants, two RH individuals). (**B**) Floral herbivore (FH) density effects on plant aboveground biomass and reproductive traits. Shown values (mean ± SE) are relative to mean FH_0_ = 0, plants with no FH (see absolute values in Supplementary Material 2, Figure S1). White bars are shown for 0 FH density (FH_0_ plants, absence of FH), light green bars are shown for low FH density (FH_1_ plants, one FH individual), dark green bars are shown for high FH density (FH_2_ plants, two FH individuals).

RH reduced aboveground biomass and flower number regardless of the herbivore density (a non-linear effect; Table 2, Figure 2A). On the contrary, an increase in RH density resulted in a greater decrease in fruit number (a linear effect; Table 2, Figure 2A). RH had no effect on seed number (Table 2, Figure 2A). FH density also affected aboveground biomass, where plants with high FH had more aboveground biomass than plants with low FH (non-linear effect; Table 2, Figure 2B). FH decreased the number of flowers, fruits, and seeds regardless of FH density (non-linear effect; Table 2, Figure 2B).

**Figure 3:**
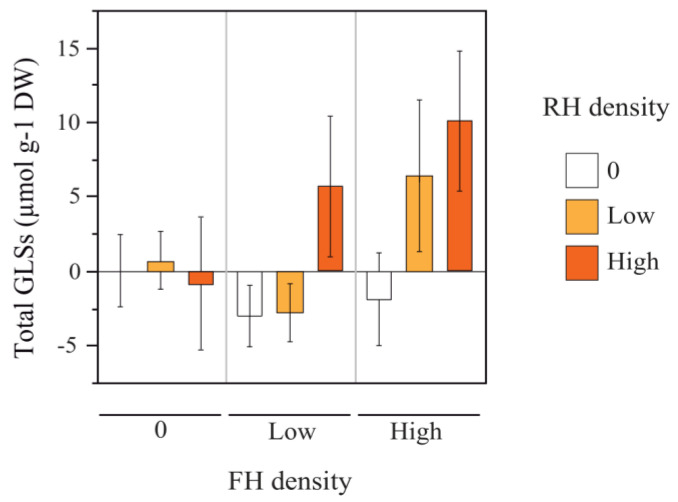
Root herbivore (RH) and floral herbivore (FH) density (0 or absence, low, and high) effects on total glucosinolate (GLS) concentrations. Shown values (mean ± SE) are relative to the focal treatment (mean RH_0_FH_0_ = 0, plants without RH and FH; see absolute values in Supplementary Material 2, Figure S1). As follows: 0 FH density = absence of FH, FH_0_ plants; low FH density = FH_1_ plants, one FH individual; high FH density = FH_2_ plants, two FH individuals_._ White bars are shown for 0 RH density (RH_0_ plants, absence of RH), orange bars are shown for low RH density (RH_1_ plants, one RH individual), red bars are shown for high RH density (RH_2_ plants, two RH individuals).

Regarding plant defense, ten glucosinolates (GLS) were identified in leaves: four indolic (0.59 ± 0.20 µmol g^−1^ of dry weight) and six aliphatic GLSs (12.36 ± 1.07 µmol g^−1^ of dry weight). Indol-3-yl-methyl GLS was the main indolic compound (0.52 ± 0.20 µmol g^−1^ of dry weight), while 3-butenyl GLS was the main aliphatic compound (11.45 ± 1.01 µmol g^−1^ of dry weight). RH and FH densities interactively affected the total leaf GLS concentrations (Table 2). Total GLS concentrations in leaves were highest when both herbivore groups were present at high density: on high RH plants, plants with high FH had higher GLS concentrations than plants without FH, and on high FH plants, plants with high RH had higher GLS concentrations than plants without RH (Figure 3). A similar pattern was found for aliphatic GLSs (Table 2). RH or FH densities did not affect total indolic GLS concentrations. We also observed no effect of RH and FH densities on C/N ratios in leaves and seeds (Table 2).

### 2.3. Herbivore Effects on FH Caterpillar Development Time

FH caterpillar development time was shortened with increasing density of RH (*χ²* = 7.54, *P* = 0.006, df = 1,38). Caterpillars developed faster on high RH plants (14.86 ± 1.07 days) than on both plants with no RH (17.27 ± 0.71 days) and low RH plants (16.03 ± 0.79 days). FH density (*χ²* = 0.09, *P* = 0.76, df = 1,38) and the interaction term of RH and FH densities (*χ²* = 1.40, *P* = 0.23, df = 1,38) had no effect on caterpillar development time.

### 2.4. Structural Equation Models (SEMs) of RH and FH Density Effects on Plant Reproduction

**Figure 4:**
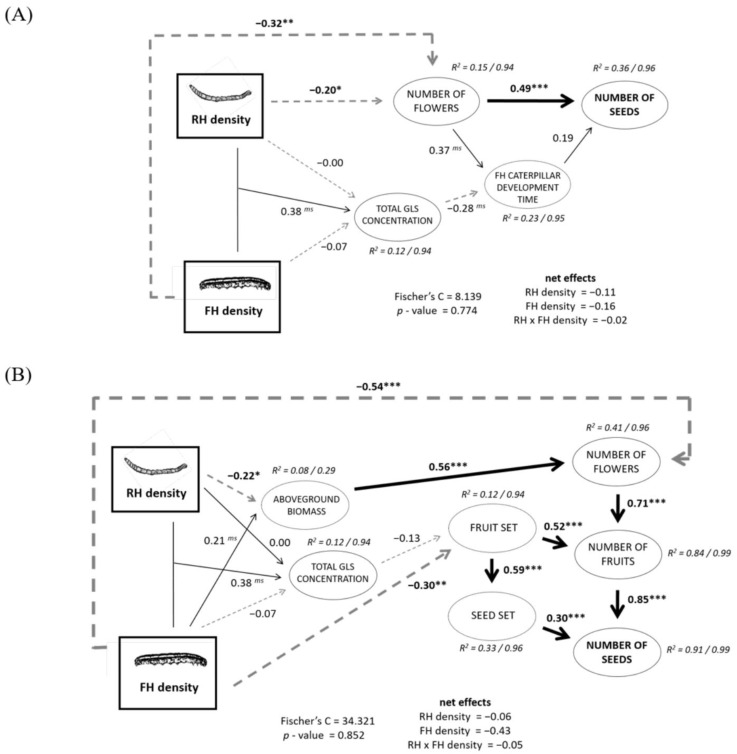
(**A**) Final piecewise SEM 1 parsing the direct, indirect, and net effects of RH and FH densities on plant reproduction (number of seeds) through FH caterpillar development time. (**B**) Final piecewise SEM 2 parsing the direct and indirect effects of RH and FH densities on sequential plant reproductive components and its net effects on plant reproduction (number of seeds). Fruit set was calculated as the proportion of flowers that passed to fruits, and seed set as the proportion of ovules that passed to seeds. For both (**A**,**B**), standardized path coefficients are shown next to each path, and their significance level is shown as ^ms^ *p* < 0.08, * *p* < 0.05, ** *p* < 0.01, *** *p* < 0.001. Solid lines denote positive and dashed lines negative relationships, and their thickness is scaled to the significance level. Variance explained by the component models (R^2^) is reported as marginal/conditional.

SEM 1 shows that the negative effect of the RH and FH on plant reproduction (Figure 2) was mainly a consequence of their respective negative effects on flower number (Figure 4A), which affected seed number both directly and indirectly by lengthening the caterpillar development time. In addition, an interactive effect of RH and FH densities on the total glucosinolate concentration affected the caterpillar development time, but the consequences from this effect on seed production were practically negligible (Figure 4A, Supplementary Material 3, Table S3). SEM 2 furthermore indicated that the effect of RH density on the number of flowers was indirect via a decreasing aboveground biomass (Figure 4B). FH density effect on number of flowers was partly direct and partly indirect through a positive effect of FH on aboveground biomass (Figure 4B). The negative consequences of FH density on seed production were also due to a direct negative effect on the fruit set (Figure 4B, Supplementary Material 4, Table S5).

### 2.5. Transgenerational Herbivore Effects on Seedling Emergence: Lifetime Fitness Estimation

**Figure 5:**
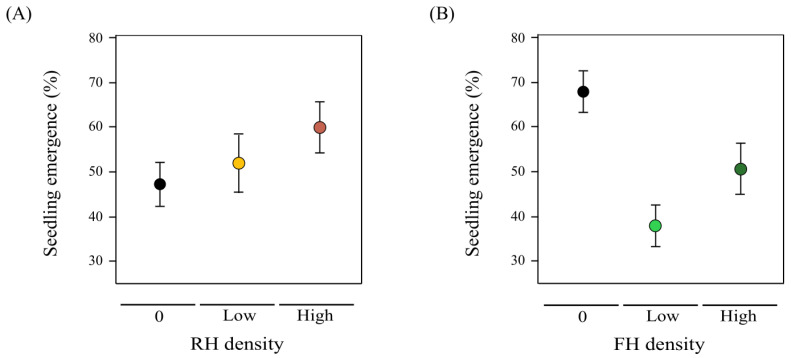
(**A**) Maternal root herbivore (RH) density effect on seedling emergence. Black points are shown for 0 RH density (RH_0_ plants, absence of RH), orange points are shown for low RH density (RH_1_ plants, one RH individual), red points are shown for high RH density (RH_2_ plants, two RH individuals). (**B**) Maternal floral herbivore (FH) density effect on seedling emergence. Black points are shown for 0 FH density (FH_0_ plants, absence of FH), light green points are shown for low FH density (FH_1_ plants, one FH individual), dark green points are shown for high FH density (FH_2_ plants, two FH individuals).

Seedling emergence was non-significantly affected by maternal RH density (*F*_1,51_ = 2.81, *P* = 0.09, Figure 5A), while it was reduced when maternal plants endured low or high FH densities (a non-linear effect; *F*_1,51_ = 5.85, *P* = 0.01, Figure 5B). The interaction term had no effect on seedling emergence (*F*_1,51_ = 0.26, *P* = 0.61).

## 3. Discussion

### 3.1. Density-Dependent FH Damage

Despite promoting a mixed resistance–tolerance response, reproductive tissue consumption by FH was detrimental for plant reproductive success, in agreement with previous studies [27,34,69]. Damage caused by FH even entailed a reduction in seedling emergence, enlarging the negative consequences of this type of herbivory [27,52]. Reduced emergence was not related to the seed nutrient content, so lower seed mass or defects in seed formation triggered by reproductive tissue consumption could be the potential mechanisms [7,27,53,60]. However, the FH negative effects on flower, fruit, and seed production and seedling emergence did not depend on the herbivore density, pointing to a non-linear relationship between herbivore density and damage for florivory. Hence, the plant seems to display an adaptive defensive phenotype that reduced the fitness costs of the florivory [7,70,71].

Previous studies suggest that plants, particularly those adapted to tolerate herbivory, reproduce at the maximum rate when challenged by a severe threat to fitness [72,73]. The tolerance response of *M. moricandioides* to high densities of FH was supported by the GEE results, showing that reproductive tissue production was boosted when FH caterpillars were actively feeding on plants (to a large extent eaten by FH caterpillars afterward, Figure 1). That the plant promoted an intense tolerance response is reinforced by FH impact on aboveground biomass, which points to differential plant responses in dependence of FH density. When enduring floral herbivory by a single caterpillar, the aboveground biomass was decreased, whereas when enduring floral herbivory by two caterpillars, plants increased aboveground biomass. In this case, higher aboveground biomass is probably a by-product of compensatory growth [74].

### 3.2. Density-Dependent RH Damage

While florivory triggered the production of reproductive tissue, our analyses showed the opposite for root herbivory. RH density had a non-linear and negative effect on aboveground biomass and the number of flowers, where the effect was similar between low and high RH densities, and a linear effect on the number of fruits. However, RH damage did not significantly affect seed yield or seedling emergence, although a trend toward a greater emergence of seedlings was observed at higher density of maternal RH. The magnitude of the RH damage usually has a higher effect on growth than on reproduction in most plant species, which suggests a widespread compensatory capacity to RH [16]. A high reproductive compensatory capacity to RH was also reported for several Brassicaceae, both *M. moricandioides* [30,52] and *Sinapis arvensis* [75,76].

High RH density also reduced the development time of the FH caterpillars. RH decreased flower number and increased the GLS concentrations, both affecting FH caterpillar development time. Our GLS measurements were performed for leaves, but we assume that plants with high GLS concentrations in leaves also have high GLS concentrations in reproductive tissue, as was suggested for several defensive compounds and plant species [77,78,79,80,81,82,83]. The SEM shows that a longer caterpillar development time slightly increased seed production, and thereby shortening caterpillar development time enlarges the negative effect of the RH density on plant reproduction, although even that does not make the RH effect significant. Possibly, the slower the caterpillar development, the lower their consumption rate and the greater the plant capacity to produce new reproductive tissue and to mature its fruits, which would prevent its ingestion by caterpillars (slow-them-down strategy, see [84]).

### 3.3. RH Consequences for FH Caterpillar Performance

From the caterpillar perspective, it seems logical that flower number and GLS concentrations to some extent determine their development. Other pierid species such as *Pieris brassicae* preferably feed on GLS-rich reproductive tissues, in which they sustain higher growth rates [78], gain more weight [49], and probably increase their survival and fitness [85]. Indeed, faster development correlates with higher pupal mass in this species [64]. For the same species, other authors suggest that shortened development time could also be due to food deprivation, and deprivation implies negative fitness consequences, such as a reduction in pupal mass [86]. Our results would be supportive of both hypotheses (Supplementary Material 5, Figure S4a,b). In this way, it has to be empirically verified whether root herbivory could have an indirect facilitative effect (*in sensu* [87]) on FH caterpillars by increasing their growth rate or whether the consequences for caterpillar performance are negative due to resource (flower number) deprivation.

### 3.4. Additiveness and Non-Additiveness in Simultaneous Root and Floral Herbivory

Our results fulfill the general prediction that the fitness impact of simultaneous herbivory at different plant parts tends to be independent [15]. There were also no consequences on the leaf and seed nutrient content, albeit herbivory on resource sources (roots) and resource sinks (reproductive tissues) can alter the source–sink relations [88]. Lower seed provisioning requirements due to a reduced seed yield in FH plants could counterbalance the resource deprivation due to tissue consumption, entailing equivalent seed nutrient contents. On the contrary, the plant defensive response to the FH and RH densities was non-additive. When the plant endured high FH compensatory growth prevailed, and only when accompanied by high root herbivory, enhanced chemical defense was also evident. These trait combinations may have resulted in a linear although non-significantly detrimental fitness impact of the RH density and in a non-linear relationship between herbivore density and fitness damage in the case of florivory.

Plants employ a series of regulatory switches to prevent the costly coexpression of high levels of growth and defense, which can be maladaptive, and must *opt* between the range of trait combinations to achieve the maximum fitness [71,89,90]. While the functionality of the tolerance response seemed undoubtedly beneficial for the plant in the present framework, the non-additive resistance response generates more uncertainties because of its effect on FH caterpillar development and due to the high photosynthetic requirement costs from the production of secondary metabolites, such as GLSs [91,92,93].

## 4. Materials and Methods

### 4.1. Study System

The experiment was conducted in 2013 at Barranco del Espartal, a semiarid open shrub-steppe located in the semiarid Guadix-Baza Basin (Granada, southeastern Spain). The climate is distinctively continental, with strong temperature fluctuations (ranging from −14 °C to up to 45 °C) and a high seasonality (hot summers, cold winters). Due to isolation by a chain of mountains, annual precipitation does usually not exceed 300 mm [30,94], severely conditioning vegetation diversity and cover.

**Figure 6:**
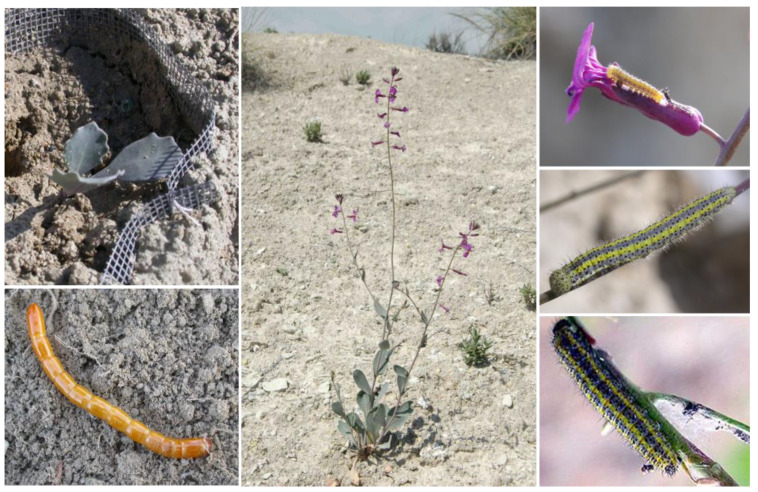
**Top left**, example of *Moricandia moricandioides* plant when moved to the field and re-potted in fiber-glass-mesh cylinders. **Bottom left**, late larval instar of *Cebrio gypsicola*. **Middle**, natural *M. moricandioides* individual in the study area. **Top right**, early larval instar of *Euchloe crameri* feeding on a *M. moricandioides* flower. **Middle right**, late larval instar of *E. crameri*. **Bottom right**, late larval instar of *Pontia daplidice* (author: José Manuel Benito Álvarez, Creative Commons BY-SA 2.5 ES).

We used the predominantly semelparous Brassicaceae species, *Moricandia moricandioides* (Boiss.) Heywood as a model system, an Iberian endemic species that inhabits semiarid areas (Figure 6, [51,62,95,96]). This herb develops as a vegetative rosette during winter and produces reproductive stalks during spring, which remain photosynthetically active during the entire season [51,74]. Flowers develop and open sequentially, and the blooming period lasts at most 3–4 weeks per individual plant, mainly between April and May [95,96,97]. Flowers remain with the stigma receptive for 3 to 5 days [74]. Fruit production begins in the first few days of flowering, ripening 20–60 seed per fruit [62,74]. After having reproduced, the vast majority of individuals die in August at the time of seed dispersal [30,51]. Similar to other Brassicaceae and related families, *M. moricandioides* produces glucosinolates as potential defense metabolites [98].

The Brassicaceae specialist caterpillars, *Pontia daplidice* L. and *Euchloe crameri* Batler (Lepidoptera: Pieridae), are among the most important aboveground herbivores on *M. moricandioides* (Figure 6, [30,51]). Ambient herbivory by *P. daplidice* and *E. crameri* is high on natural *M. moricandioides* plants in the study area; 1.4 ± 0.1 (mean ± SE) caterpillars of these species per plant were counted in samplings carried out in the study area from 2008 to 2018. The caterpillars of both pierid species feed on reproductive tissue (floral buds, flowers, and immature fruits), have equivalent development times, and cause similar type of damage on plants, which sometimes even implies total flower and fruit consumption of the plant [51]. The most abundant root herbivore on *M. moricandioides* is *Cebrio gypsicola* Graells (Coleoptera: Cebrionidae), with an average density of 0.95 ± 0.2 larva/plant on individual root samplings (Figure 6, [30]). Both types of herbivores (root and floral herbivores) are capable of altering the production of glucosinolates [51].

### 4.2. Experimental Set-Up

We manipulated the root herbivore and floral herbivore densities in a full factorial design with two factors (root and floral herbivore densities). The root herbivore (RH) density was manipulated at three levels: control plants with no root herbivores (RH_0_, absence), treatments with one root herbivore individual (RH_1_, low), and treatments with two root herbivore individuals (RH_2_, high). Floral herbivore (FH) density was likewise varied with three levels: control plants with no floral herbivores (FH_0_, absence), treatments with one floral herbivore individual (FH_1_, low), and treatments with two floral herbivore individuals (FH_2_, high). These density gradients for both root and floral herbivores are realistic based on observations of *M. moricandioides* in the study area.

The experiment was set up on 14 March 2013, when we moved the *M. moricandioides* seedlings to the study site. These plants came from seeds collected from the study area during the autumn of 2012 and were grown with soil from the study area in a common garden. In the field, we transplanted 108 experimental plants in each of 6 blocks (2 replicates × 9 treatments/block), where plants were 30 cm apart from each other. None of the plants had a reproductive stem when being transplanted to the field. In the absence of natural rain, all plants were watered and net-covered during the first week in the field to ensure their establishment.

To set up the RH treatments, plants were re-potted when moved to the field using mixed macroarthropod-free soil from the study site. The pots consisted of fiber-glass-mesh cylinders (15 × 20 cm) of 1 mm mesh size to inhibit the entrance or escape of belowground macroinvertebrates (Figure 6). These pots were then buried with the upper surface level with the ground. The reliability of this methodology in recording root herbivory effects was previously demonstrated in this system, as ~90% of the larvae can be recovered from the pots at the time of plant harvest [51]. Once the plants were established in the field, third-instar *C. gypsicola* larvae, collected in the study area during winter 2012–spring 2013, were added to plants assigned to RH_1_ and RH_2_ treatments (25 March 2013). 

To set up the FH treatments, we removed all *P. daplidice* and *E. crameri* eggs from FH_0_ plants but allowed natural oviposition by these species on reproductive stalks of FH_1_ and FH_2_ plants. In cases where no caterpillars had hatched on FH_1_ or FH_2_ plants when plants already had reproductive tissues (n = 7), first instar caterpillars collected from the study area were added. Once FH_1_ or FH_2_ plants had the designated FH density level, additional pierid eggs laid by butterflies were thereafter removed from these plants. When caterpillars died or disappeared before completing their larval cycle and moving to pupate, we replaced them by adding same-instar caterpillars collected from the study area to the plants.

Twenty plants were excluded from analysis because they died during the experiment (n = 3), did not produce reproductive stalks (n = 8), or because caterpillars left the plant before completing their larval cycle (n = 9). The final sample size was 88, and the sample size per treatment was RH_0_FH_0_ n = 11, RH_0_FH_1_ n = 11, RH_0_FH_2_ n = 9, RH_1_FH_0_ n = 12, RH_1_FH_1_ n = 10, RH_1_FH_2_ n = 8, RH_2_FH_0_ n = 9, RH_2_FH_1_ n = 10, and RH_2_FH_2_ n = 8.

### 4.3. Data Collection

Plant reproductive traits (number of floral bud groups, flowers, and immature fruits) were recorded on each experimental plant 3 times per week after the set-up of the experiment (from 27 March 2013, two days after the addition of RH larvae) until the end of the experiment (26 July 2013), resulting in 40 surveys. Through these records from March to July, we were able to estimate the sequential occurrence in ephemeral and immature reproductive traits per individual plant. These reproductive traits were then calculated over the entire reproductive period (from the first floral bud production until final fruit number for floral bud groups) as well as for two subperiods (from the first floral bud production until no undeveloped floral bud groups remained for flowers and from the first fruit production until final fruit number for fruits). At the end of the experiment, we counted the total number of flowers and fruits produced by each plant. All fruits were collected after complete maturation of seeds but before seed dispersal, and the number of viable seeds in each fruit was counted to quantify total seed production per plant (simplifying, number of seeds).

During the experiment, other insect herbivore abundance, pollinator visitation, and FH caterpillar parasitism rate were recorded, as well as pre-dispersal seed predator incidence in fruits; none of these variables was significantly affected by the experimental treatments (Supplementary Material 6, Table S6). We also calculated caterpillar development time (days) strictly for those caterpillars that completed their entire larval cycle without being replaced on the experimental plants (n = 53 caterpillars on n = 45 plants). When development time of both caterpillars on FH_2_ plants could be measured, we used mean value per plant.

The entire aboveground tissues were collected and dried at 40 °C for 48 h to determine aboveground dry biomass and C/N ratio of leaf tissue. C/N ratio was also determined from seeds, in both cases with a CHN Elemental Analyzer. Belowground tissues were not collected because we wanted to estimate resprouting rate at the next season. Resprouting rate was low (12.5%), and we observed no differences among treatments (data not shown).

To quantify glucosinolate (GLS) concentrations in leaves, we collected the youngest leaf of one stem of each experimental plant at the end of June, when plants had interacted with respective herbivores for multiple weeks but prior to leaf senescence. Leaves were immediately frozen and freeze-dried. The dried material was ground and extracted three times in 80% methanol after the addition of *p*-hydroxybenzyl GLS (sinalbin) used as an internal standard. GLS extraction and conversion to desulfoglucosinolates were performed following previously established methodology using high-performance liquid chromatography coupled with a diode array detector. Desulfoglucosinolates were identified by comparison of UV spectra and retention times to those identified in earlier studies [30,51,62,74]. Peaks were integrated at 229 nm and response factors of 1 for aliphatic and 0.26 for indolic GLSs were considered and related to the internal standard (response factor 0.5) and sample dry mass for calculation of concentrations.

To estimate seedling emergence on offspring, we planted 30 seeds from each of 55 experimental plants (1644 seeds in total) in autumn (between 29-September and 3-October). Seeds were planted in black peat moss in 11 × 20 seedbeds at a greenhouse with natural temperature and photoperiod conditions and protected from herbivory by a 250 μm size antithrip mesh. Seedbeds were rotated in the same direction every two days to avoid possible location effects. We supervised seedling emergence every other day from the planting day until the end of November (last observed emergence occurred on 7-November).

### 4.4. Statistical Analyses

First, generalized estimating equation models (GEEs) were performed to test the effects of each continuous factor (RH and FH densities) and their interaction over time on plant reproductive development (number of floral bud groups, flowers, and immature fruits). It was argued that plant reproduction is a hierarchical process with ephemeral structures (e.g., floral buds and flowers) that impede the assessment of florivory impact on plants [33,99]. Considering that plant tolerance responses toward florivory may involve changes in the timing and the amount of reproductive tissue produced, we used GEE models, which handle dependent observations in the same individual with a fitted correlation structure to identify how the different treatments affected the reproductive process. GEEs, i.e., the marginal modeling approach, is a powerful and pragmatic tool for analyzing correlated data and can handle non-normal distribution and heteroscedasticity [100,101].

In GEEs, we used different time lapses for the number of floral bud groups, flowers, and immature fruits (see Data Collection section). RH density was constant per individual plant over time, while we adjusted FH density to the interval of dates in which the caterpillars were present on FH_1_ and FH_2_ plants (absence of FH before and after). We tested different distributions and correlation structures for each variable using the Quasi Information Criterion (QIC) model fit, as it works well selecting the correlation structure in non-likelihood-based methods, such as GEE [102]. Floral bud groups and fruits were modeled with Poisson distribution and an autoregressive model of the 1st-order correlation structure was used due to strong correlation between following surveys. In an autoregressive model, the correlation declines with the distance between observations. Flowers were modeled with a Gaussian distribution and an exchangeable correlation structure, as there was no strong correlation between surveys. An exchangeable model has a single correlation parameter identical for all pairs of measurements on the same individual, irrespective of how far in time the measurements are from each other. These analyses were performed using R [103], with the package *geepack* [100].

Second, general or generalized linear mixed models (GLMMs) and generalized additive mixed models (GAMMs) were performed to test the effects of each continuous factor (RH and FH densities) and their interaction on plant aboveground biomass, quality (C/N ratio in leaves and seeds and GLS concentrations in leaves), and reproduction (number of flowers, fruits, and seeds). These models were also performed to test the effects of RH and FH densities and their interaction on FH caterpillar development time. Variables were modeled with Gaussian, Gamma, or Poisson distributions and were transformed when necessary (see Supplementary Material 1, Table S1 for best models for each variable in detail). Models with Gamma distribution were analyzed with inverse link function and those with Poisson distribution were analyzed with log link function. Block was included as random factor for all the variables. When overdispersion was observed (Supplementary Material 1, Table S1), models with observation-level random effects were run, which allowed for variation at plant level [104]. For each variable, we used the same distribution and random structure in GLMMs and GAMMs and compared the model fit according to Bayesian Information Criterion (BIC), which tends to favor the most parsimonious models [105]. By comparing the fit of the GLMM and the GAMM, we got a good indication whether the effects were linear or non-linear. In the case of seedling emergence, similar models with no random factor were performed to test RH and FH density effects on mean seedling emergence per mother plant. GLMMs were performed with the R package *lme4* [106] and GAMMs with the package *mgcv* [107].

Lastly, sets of component models were combined within a piecewise structural equation modeling (SEM) framework to parse the direct and indirect effects of RH and FH densities (as continuous) on plant reproduction (number of seeds) through FH caterpillar development time (SEM 1, simplified model) and through sequential plant reproductive components (SEM 2, more complex model). The SEMs were fitted using the R package *piecewiseSEM* [108]. These models allow formulating hypotheses on pathways of interaction between parameters in the model, where all parameters could act as both predictor and response variables. As recommended, the SEMs had at least 10 times as many observations as variables [109]. Variables were standardized (mean = 0, SD = 1) and we fitted the component models of the piecewise SEM as linear mixed models. For all component models, the random structure was the one used in GLMMs/GAMMs. We started with initial SEMs (for which hypothesized pathways were based on prior GLMM/GAMM results; Supplementary Material 3, Figure S3; Supplementary Material 4, Figure S4) and improved them through the stepwise procedure by evaluating Akaike’s information criterion corrected for small sample sizes (AICc) [108,109], until we procured the final SEMs with the lowest AICc scores (Supplementary Material 3, Table S2; Supplementary Material 4, Table S4). In piecewise SEM, the optimization procedure is based on the removal of irrelevant paths and the inclusion (based on Shipley’s test of *d*-separation) of any of the non-hypothesized biologically relevant paths that can improve the model [108,109]. In the SEMs, paths with marginally significant effects were considered to be of sufficient biological relevance, so they were contemplated as such.

## 5. Conclusions

Our results with *M. moricandioides* provide evidence that plants prioritize differing trait combinations depending on the herbivore density both below- and aboveground to achieve maximum (or minimize impact on) fitness. The optimal combination of resistance and tolerance would thus vary according to the type of herbivory, the herbivore density, and the type of herbivory × density interaction. Therefore, we encourage the performance of more comparable studies, in which the density-dependent effect of several herbivores is simultaneously studied, and its relationship with the tolerance, resistance, and fitness of the plant is evaluated. Finally, our findings claim to consider a temporally-explicit approach when analyzing the effects of florivory on plant defense and reproduction, given the ephemerality of the consumed reproductive tissues and thus of florivory itself. A complete survey of plant reproductive traits throughout their reproduction may allow a better quantification of the plant defensive responses to these herbivores and the direct and indirect effects of florivory.

## Figures and Tables

**Table 1:** Results of generalized estimating equation models (GEEs) for the effect of root herbivores (RH) and floral herbivores (FH) on plant reproductive traits development over time. Significant values (*p* < 0.05) are highlighted in bold.

	RH				FH				RH × FH			
Variable	*df*	*χ²*	*z*	*p*	*df*	*χ²*	*z*	*p*	*df*	*χ²*	*z*	*p*
Floral bud group production	1,1704	**4.14**	**−2.05**	**0.04**	1,1704	0.03	0.16	0.86	1,1704	0.12	−0.34	0.73
Flower production	1,1389	0.50	−0.70	0.47	1,1389	**11.00**	**3.31**	**<0.0001**	1,1389	0.33	0.57	0.56
Fruit production	1,1202	**8.15**	**−2.85**	**0.004**	1,1202	**76.89**	**8.76**	**<0.0001**	1,1202	2.03	1.42	0.15

**Table 2:** GLMM/GAMM results for the effect of root herbivores (RH) and floral herbivores (FH) on plant aboveground biomass, reproductive traits, leaf and seed nutrient content, and leaf glucosinolates (GLSs). Significant values (*P* < 0.05) are highlighted in bold.

			RH			FH			RH × FH		
	GLMM/GAMM	*F/χ*²	*df*	Statistic	*p*	*df*	Statistic	*p*	*df*	Statistic	*p*
*Aboveground biomass* *and reproduction*											
Aboveground biomass	GLMM	*χ²*	1,73	**6.54**	**0.03**	1,73	**3.92**	**0.04**	1,73	0.01	0.92
Number of flowers	GAMM	*χ²*	1,79	**5.15**	**0.02**	1,79	**8.89**	**0.002**	1,79	0.15	0.69
Number of fruits	GAMM	*χ²*	1,79	**5.05**	**0.02**	1,79	**18.22**	**<0.0001**	1,79	0.03	0.85
Number of seeds	GAMM	*χ²*	1,79	2.17	0.14	1,79	**12.05**	**0.0005**	1,79	0.26	0.60
*Leaf and seed nutrient content*											
C/N ratio in leaves	GLMM	*χ²*	1,76	1.10	0.29	1,76	0.43	0.51	1,76	0.00	0.92
C/N ratio in seeds	GLMM	*χ²*	1,59	0.04	0.82	1,59	2.04	0.15	1,59	0.44	0.50
*Leaf glucosinolates (GLSs)*											
Total GLSs	GLMM	*F*	1,77	**4.62**	**0.03**	1,77	1.16	0.28	1,77	**4.79**	**0.03**
Aliphatic GLSs	GLMM	*F*	1,77	**5.08**	**0.02**	1,77	1.36	0.24	1,77	**4.03**	**0.04**
Indolic GLSs	GLMM	*F*	1,77	2.65	0.10	1,77	0.33	0.56	1,77	0.28	0.59

## Data Availability

The data presented in this study are available on request from the corresponding authors. The data will be published in Figshare.

## Supplementary Materials

The following supporting information can be downloaded at: https://www.mdpi.com/article/10.3390/plants12020283/s1. Supplementary material 1, Table S1: Model structure and fit of GLMMs and GAMMs. Supplementary material 2, Figure S1: Plots with absolute values for analyzed variables. Supplementary material 3, Table S2: SEM 1 stepwise selection procedure. Supplementary material 3, Table S3: SEM 1 standardized direct, indirect and total size effects. Supplementary material 3, Figure S2: Initially hypothesized SEM 1. Supplementary material 4, Table S4: SEM 2 stepwise selection procedure. Supplementary material 4, Table S5: SEM 2 standardized direct, indirect and total size effects. Supplementary material 4, Figure S3: Initially hypothesized SEM 2. Supplementary material 5, Figure S4: Relationship between FH caterpillar development time, glucosinolate concentrations and flower number. Supplementary material 6, Table S6: GLMM/GAMM results for the effect of RH and FH on insect herbivore abundance, pollinator visitation and FH caterpillar parasitism rate.
