# Supplementary material for: Density-Dependent Effects of Simultaneous Root and Floral Herbivory on Plant Fitness and Defense
Source: Plants (Basel). 2023 Jan 7;12(2):283. doi: 10.3390/plants12020283 (PMC9867048; doi:10.3390/plants12020283)
Supplement: Supplementary file 1 [file plants-12-00283-s001.zip › plants-2087928-supplementary.pdf]

Density-dependent effects of simultaneous root and floral herbivory on plant fitness and defense

Martin Aguirrebengoa [1,*], Caroline Müller [2],

Peter A. Hambäck [3] and Adela González-Megías [1,*]

SUPPLEMENTARY MATERIAL

SUPPLEMENTARY MATERIAL 1

Table S1. Model structure and fit of GLMMs and GAMMs for analyzed variables.

Variable	Distribution	Random structure	BIC GLMM	BIC GAMM
FH caterpillar development time	Gamma	Block<br>Plant	-519.829	-241.882
Aboveground biomass	Gamma	Block	10.030	304.303
Number of flowers	Poisson	Block<br>Plant	747.763	200.073
Number of fruits	Poisson	Block<br>Plant	575.836	250.446
Number of seeds	Poisson	Block<br>Plant	1141.135	436.815
C/N ratio in leaves	Gamma	Block<br>Plant	-756.116	-559.616
C/N ratio in seeds	Gamma	Block<br>Plant	-851.526	-413.336
Total GLSs*	Gaussian	Block<br>Plant	256.451	257.186
Aliphatic GLSs*	Gaussian	Block<br>Plant	256.304	257.294
Indolic GLSs*	Gaussian	Block<br>Plant	264.172	264.172
Seedling emergence	Gaussian	-	0.822	0.822

* GLS concentrations are range/(n-1) transformed

SUPPLEMENTARY MATERIAL 2

Figure S1. Locally estimated scatterplot smoothing (LOESS) plots for analyzed plant variables regarding root herbivore (RH) density, floral herbivore (FH) density, and both herbivore group densities.

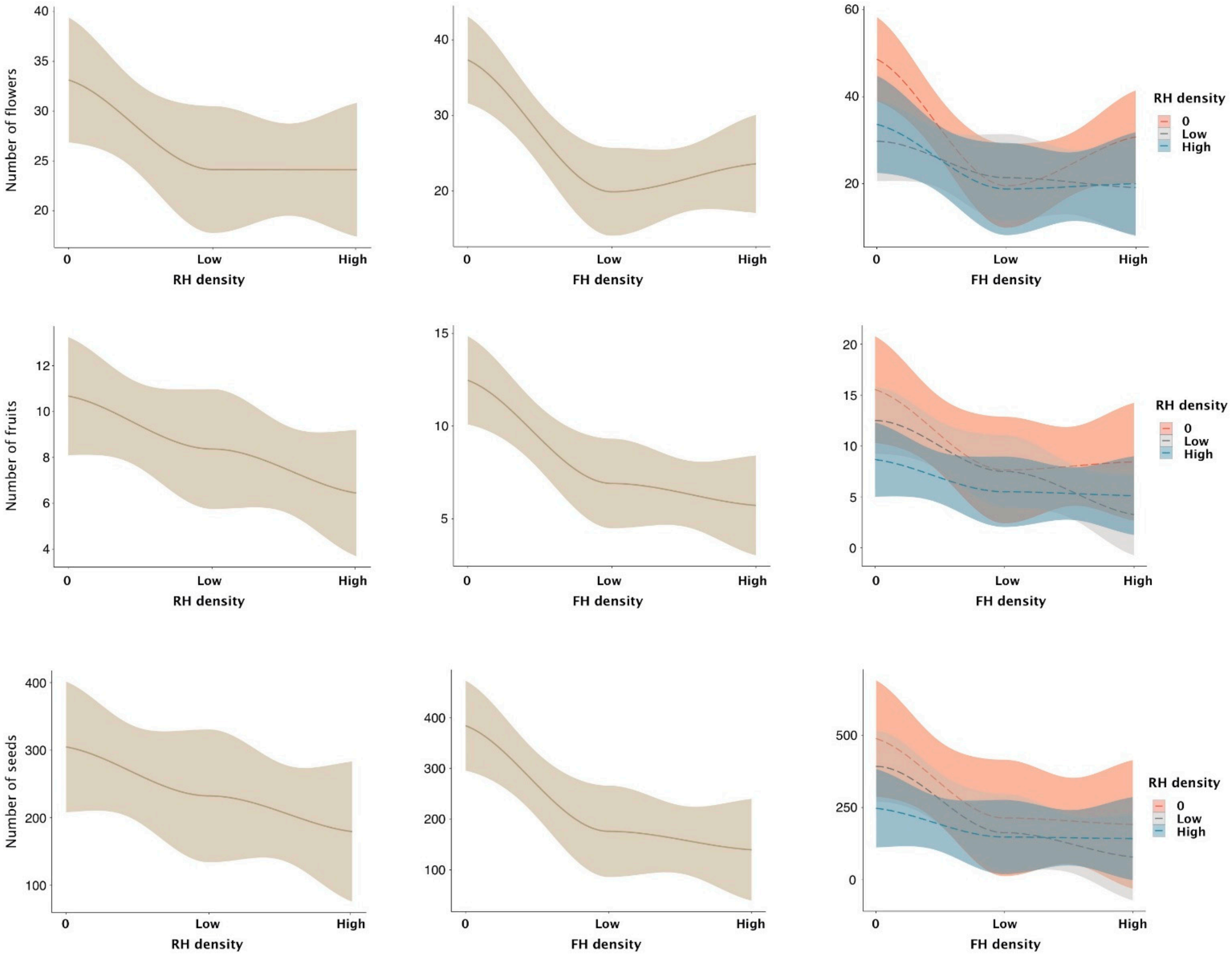

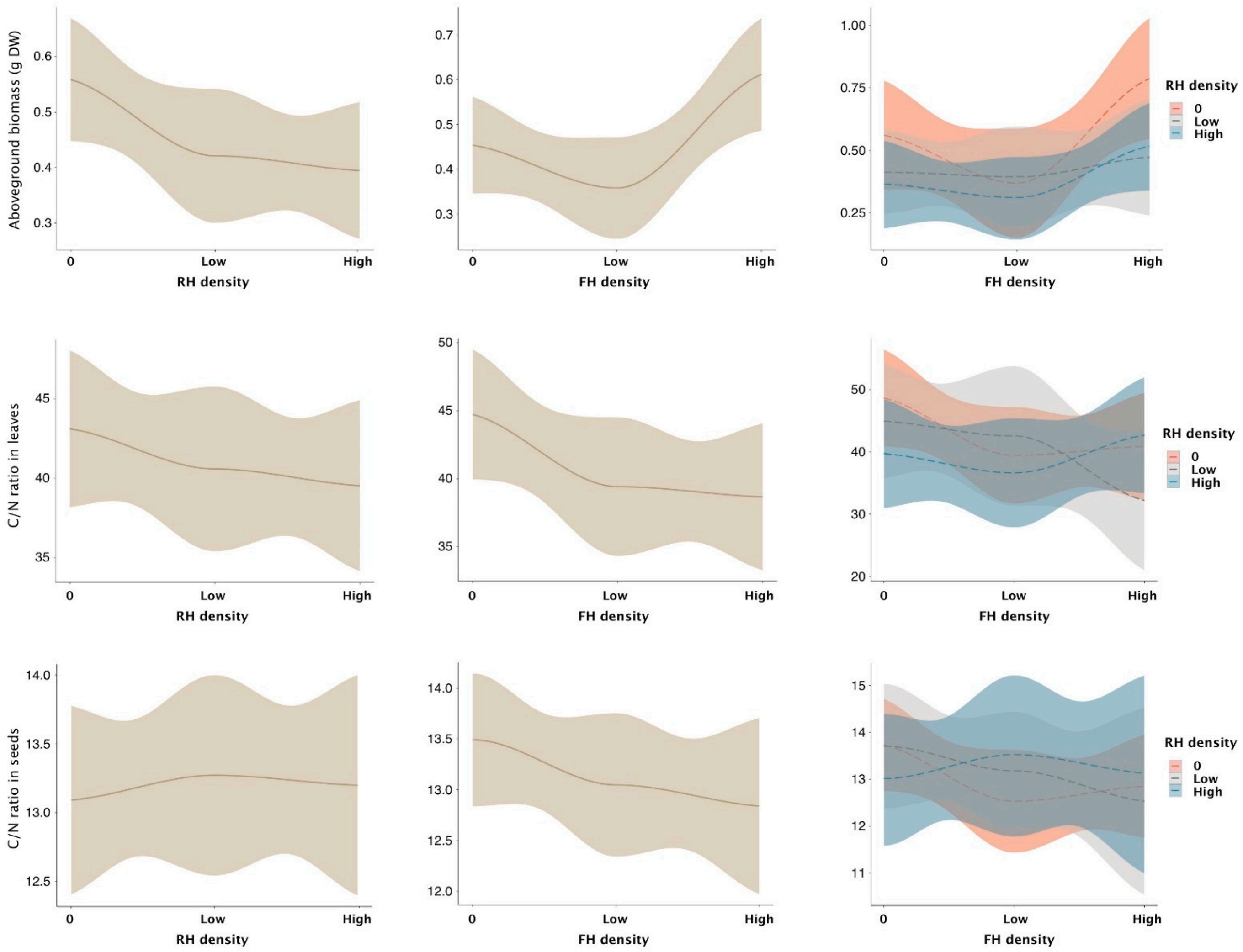

Aboveground biomass (g DW)
C/N ratio in leaves
C/N ratio in seeds
RH density
FH density
0
Low
High

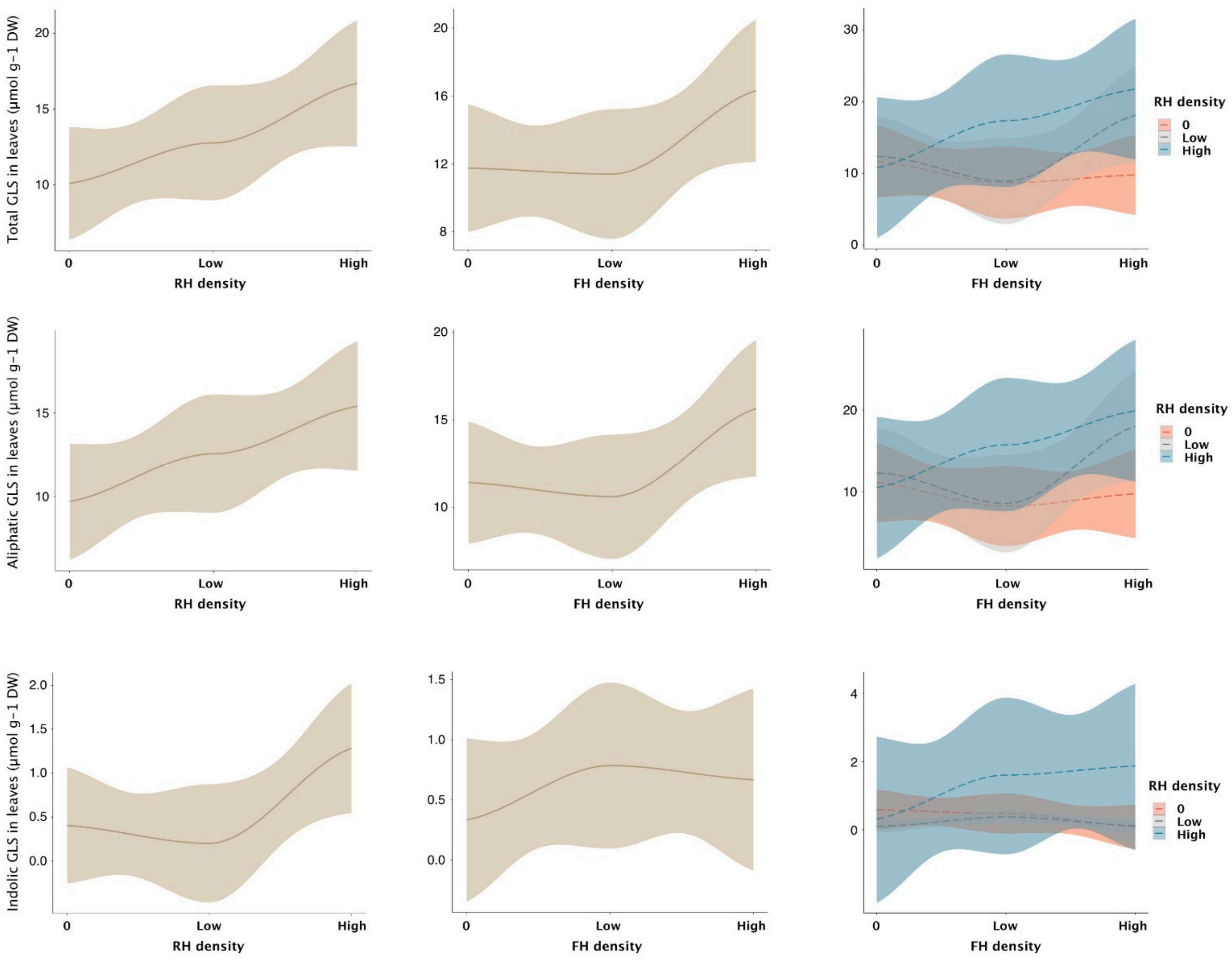
Total GLS in leaves (µmol g-1 DW)
Aliphatic GLS in leaves (µmol g-1 DW)
Indolic GLS in leaves (µmol g-1 DW)
RH density
FH density
0
Low
High
RH density
0
Low
High

SUPPLEMENTARY MATERIAL 3

Table S2. Stepwise SEM selection procedure for RH and FH density effects through FH caterpillar development time on plant seed production (SEM 1).

Model	Removed paths	Added paths	AICc	ΔAICc	df	Fischer's C	*P*
Full initial model			172.785	25.728	10	6.274	0.792
model 2	FH density → Number of seeds	-	161.105	14.048	12	7.946	0.789
**model 3**	Total GLS concentration x Number of flowers (interaction) → Seed set	-	**147.057**	**-**	**12**	**8.139**	**0.774**

Table S3. Standardized direct, indirect and total size effects of RH density, FH density and their interaction in the final SEM 1.

Cause	Effects on	Direct	Indirect	Total
RH density	Number of flowers	-0.20	-	-0.20
	Total GLSs	-0.00	-	-0.00
	FH caterpillar development time	-	-0.07	-0.07
	**Number of seeds**	**-**	**-0.11**	**-0.11**
FH density	Number of flowers	-0.32	-	-0.32
	Total GLSs	-0.07	-	-0.07
	FH caterpillar development time	-	-0.09	-0.09
	**Number of seeds**	**-**	**-**	**-0.16**
RH x FH densities interaction	Number of flowers	-	-	-
	Total GLSs	0.38	-	0.38
	FH caterpillar development time	-	-0.10	-0.10
	**Number of seeds**	**-**	**-0.02**	**-0.02**

Figure S2. Initially hypothesized SEM for RH and FH density effects through FH caterpillar development time on plant seed production (SEM 1). Solid lines denote positive and dashed lines negative relationships.

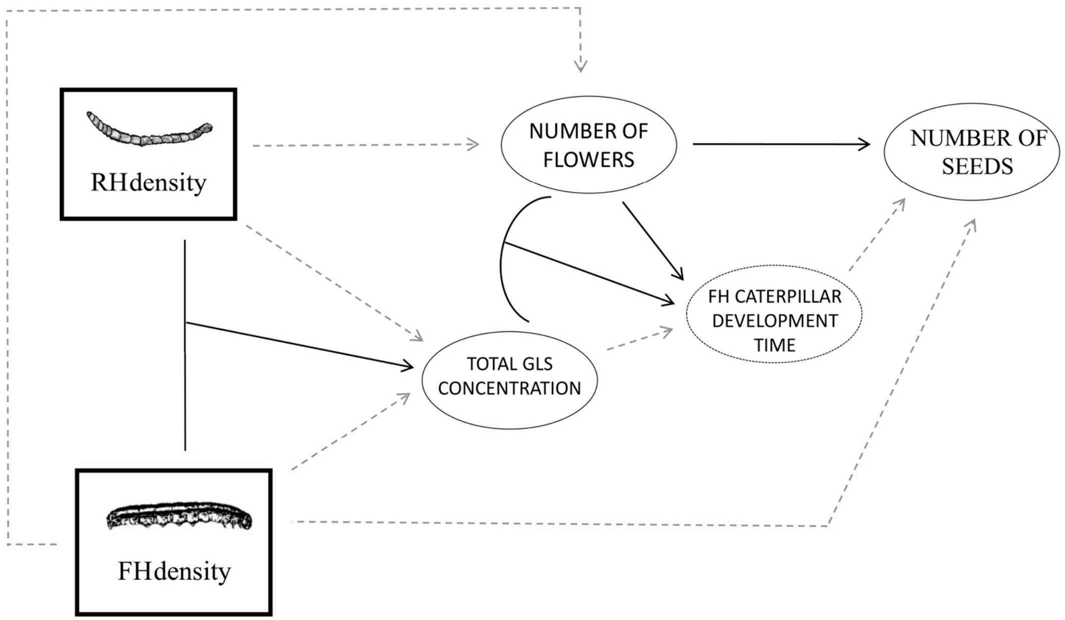

SUPPLEMENTARY MATERIAL 4

Table S4. Stepwise SEM selection procedure for RH and FH density effects on sequential plant reproductive components and seed production (SEM 2).

Fruit set was calculated as the proportion of flowers that passed to fruits, and seed set as the proportion of ovules that passed to seeds.

Model	Removed paths	Added paths	AICc	ΔAICc	df	Fischer's C	*P*
Full initial model			455.673	199.767	34	57.721	0.007
model 2	Aboveground biomass → Fruit set<br>Number of flowers → Fruit set<br>Aboveground biomass → Seed set<br>Total GLS concentration → Seed set	-	309.762	53.856	42	52.778	0.123
model 3	RH density → Number of flowers	-	302.531	46.625	44	55.514	0.114
model 4	Total GLS concentration → Fruit set	-	302.591	46.685	46	61.471	0.063
model 5		Total GLS concentration → Fruit set<br>Fruit set → Seed set	264.370	8.464	42	32.735	0.847
**model 6**	FH density → Seed set	-	**255.906**	**-**	**44**	**34.321**	**0.852**
model 7	-	Total GLS concentration → Number of flowers	257.300	1.394	42	29.613	0.925
model 8	Total GLS concentration → Number of flowers	Number of flowers → Number of seeds	280.685	24.779	42	39.939	0.562
model 9	Number of flowers → Number of seeds	Fruit set → Number of seeds	281.145	25.239	42	40.142	0.553

Table S5. Standardized direct, indirect and total size effects of RH density, FH density and their interaction in the final SEM 2.

**Cause**	**Effects on**	**Direct**	**Indirect**	**Total**
RH density	Aboveground biomass	-0.22	-	-0.22
	Total GLSs	0.00	-	0.00
	Number of flowers	-	-0.12	-0.12
	Fruit set	-	-0.00	-0.00
	Number of fruits	-	-0.08	-0.08
	Seed set	-	-0.00	-0.00
	**Number of seeds**	**-**	**-0.06**	**-0.06**
FH density	Aboveground biomass	0.21	-	0.21
	Total GLSs	-0.07	-	-0.07
	Number of flowers	-0.54	0.11	-0.43
	Fruit set	-0.30	0.01	-0.29
	Number of fruits	-	-0.45	-0.45
	Seed set	-	-0.17	-0.17
	**Number of seeds**	**-**	**-0.43**	**-0.43**
RH x FH densities interaction	Aboveground biomass	-	-	-
	Total GLSs	0.38	-	0.38
	Number of flowers	-	-	**-**
	Fruit set	-	-0.05	-0.05
	Number of fruits	-	-0.02	-0.02
	Seed set	-	-0.03	-0.03
	**Number of seeds**	**-**	**-0.05**	**-0.05**

Figure S3. Initially hypothesized SEM for the direct and indirect RH and FH density effects on sequential plant reproductive components and seed production (SEM 2). Fruit set was calculated as the proportion of flowers that passed to fruits, and seed set as the proportion of ovules that passed to seeds.

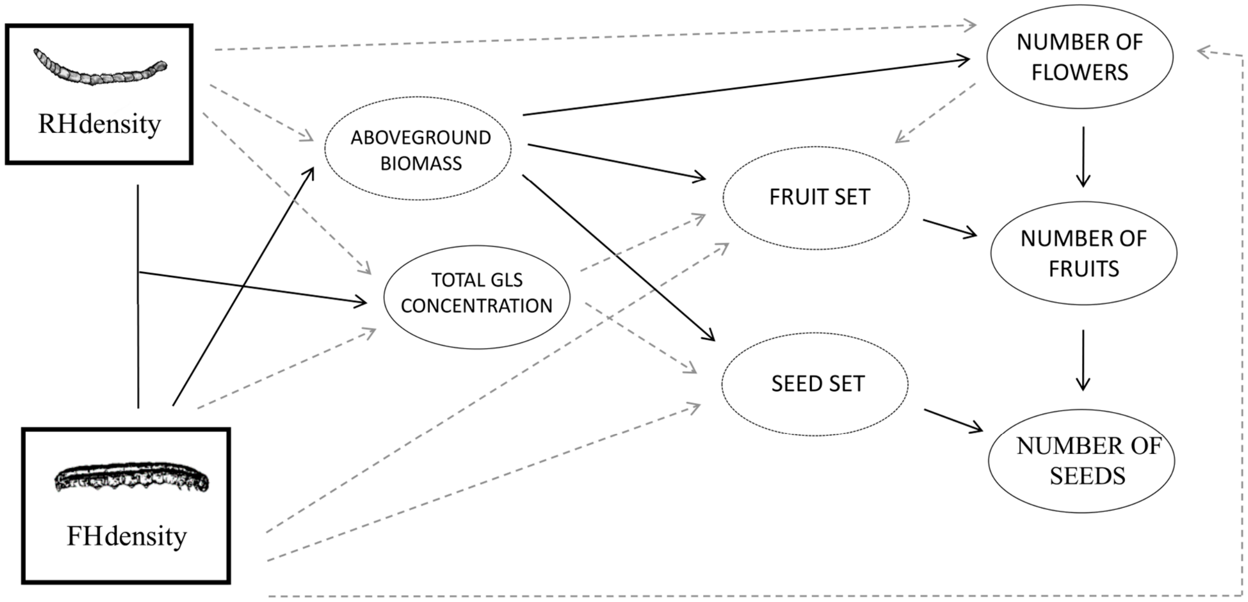

SUPPLEMENTARY MATERIAL 5

Figure S4. (A) Linear and quadratic regressions between the number of days for caterpillar development and aliphatic GLS concentration (plants with < 35 µmol aliphatic GLS g^{-1} of dry weight), indolic GLS concentration (plants with < 8 µmol indolic GLS g^{-1} of dry weight) and number of flowers. When development time for both caterpillars in FH_2 plants could be measured, mean value per plant is shown. (B) 3D and 2D planes of the interaction between total GLS concentration (plants shown in A) and number of flowers on the number of days for caterpillar development.

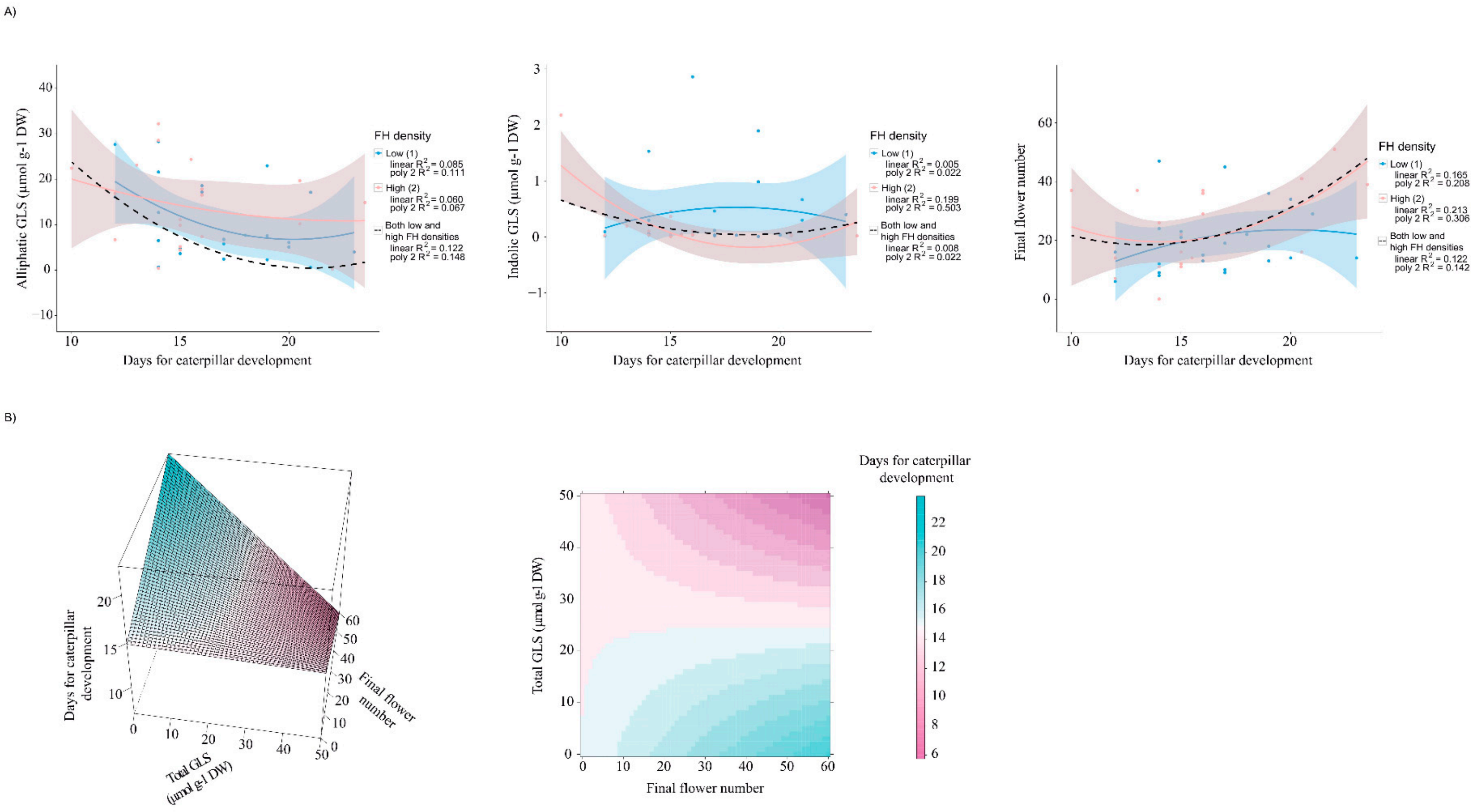

SUPPLEMENTARY MATERIAL 6

Data collection

During the study period, several species of herbivorous insects visited and fed on the experimental plants. To score the abundance of free-living herbivorous insects, the number of naturally occurring individuals was recorded on each experimental plant 3 times per week after the set-up of the experiment, giving a total of 40 surveys. In the case of aphids, we subtracted at each census the number of individuals of the same instar/type (winged versus not winged) counted in the previous census to avoid problems of summing individuals twice. Total abundance of each herbivore species or family was calculated by summing the number of individuals recorded during all the surveys. Herbivores were in addition assigned to their corresponding guild.

A total of 3.65 ± 0.90 (mean ± SE) suckers per plant were observed, which in its great majority belonged to 3 different taxonomic families: 2.10 ± 0.87 Aphididae (more than half of them belonging to the generalist species *Aphis fabae*, followed by the specialist species *Brevicoryne brassicae* and *Lipaphis erysimi*, with few individuals of the generalist species *Myzus persicae)*, 0.46 ± 0.09 Cicadellidae and 0.34 ± 0.08 Issidae (mainly *Agalmatium bilobum*). 1.11 ± 0.19 chewers per plant were also noted, which mostly belonged to larval stages of three specialist species: 0.62 ± 0.13 *Plutella xylostella* (Lepidoptera: Plutellidae), 0.19 ± 0.05 *Tenthredo sebastiani* (Hymenoptera: Tenthredinidae) and 0.19 ± 0.07 *Hellula sp.* (Lepidoptera: Cambridae). We also found 0.14 ± 0.04 leaf miners per plant (only present in 12 experimental plants). In addition, all fruits were checked for pre-dispersal seed predators when collected, and pre-dispersal seed predator presence/absence on plants was noted.

To record the abundance of pollinators, 14 censuses were carried out at midday during the peak of the flowering season (from 6-May to 13-June). These censuses consisted of 5 minutes observation of

all plants in each block, noting the identity and number of flower contacts made by each pollinator, as well as the number of open flowers per plant. The abundance of pollinators was calculated using two variables, which provide complementary information in terms of the quantity and quality of pollen supply: 1) Number of individuals, calculated as the mean number of individual insects visiting a flower, and 2) Number of contacts, calculated as the mean number of visits per flower.

1.22 ± 0.07 pollinator contacts and 0.84 ± 0.03 individuals per open flower were recorded during pollinator censuses, on 516 available flowers of 79 plants (mean ± SE: 3.35 ± 0.20 pollinator censuses per plant, with 1.86 ± 0.20 flowers per plant/census). Pollinator assemblage was mainly represented by large-tongued Antophoridae bees (62%), medium-tongued Halictidae bees (14%), small-tongued Apidae (*Ceratina*) bees (8%), bee flies (10%) and pierid butterflies (2%).

The incidence of caterpillar parasitism was calculated for n = 40 caterpillars on n = 34 plants. *Cotesia kazak* Telenga (Braconidae: Hymenoptera) was the only occurring parasitoid species during the experiment, and parasitized both *Pontia daplidice* and *Euchloe crameri* caterpillars. *Cotesia kazak* is a koinobiont endoparasitoid and can parasitize first instar caterpillars of these species, thus only those caterpillars that hatched on the experimental plants were considered. In addition, caterpillars had to stay on plants at least until late-third instar to be considered, because it is when *C. kazak* larvae hatch from the host and can be confirmed if caterpillars were parasitized or not.

Statistical analyses

General and generalized linear mixed models (GLMMs) and generalized additive mixed models (GAMMs) were performed to test the effects of each continuous factor (RH and FH densities) and their interaction on response variables, as detailed in the main text.

Results

RH and FH densities had no effect on insect herbivore abundance, pollinator visitation and FH caterpillar parasitism rate (Table S6), not even when (herbivores and pollinators) were analyzed at the taxonomic level or when analyzed (herbivores) in terms of the degree of specialization (data not shown).

Table S6. GLMM/GAMM results for the effect of root herbivores (RH) and floral herbivores (FH) on insect herbivore abundance, pollinator visitation and FH caterpillar parasitism rate.

					**RH**		**FH**		**RH x FH**	
	Distribution	Random structure	GLMM/ GAMM	F / χ^2	Statistic	P	Statistic	P	Statistic	P
Sucker abundance	Poisson	Block Plant	GLMM	χ^2	2.45	0.11	0.17	0.67	1.48	0.22
Chewer abundance	Negative binomial	Block	GLMM	χ^2	2.36	0.12	0.00	0.97	2.47	0.11
Pre-dispersal seed predator incidence	Binomial	Block	GLMM	χ^2	3.15	0.07	0.33	0.56	0.00	0.98
Pollinator contacts	Gaussian	Block	GAMM	F	0.32	0.57	0.14	0.70	0.06	0.80
Pollinator individuals	Gaussian	Block	GAMM	F	2.45	0.12	0.77	0.38	1.38	0.24
Parasitoid attack rate	Gaussian	Block	GAMM	F	0.79	0.38	0.53	0.47	0.42	0.51
